# Impact of pre-transplant time on dialysis on survival in patients with lupus nephritis

**DOI:** 10.1007/s10067-018-4115-1

**Published:** 2018-05-11

**Authors:** Eleana Ntatsaki, Alba Velo-Garcia, Vassilios S. Vassiliou, Alan D. Salama, David A. Isenberg

**Affiliations:** 1 Centre for Rheumatology, Division of Medicine, University College London, 250 Euston Road, London, NW1 2PG UK; 2 Rheumatology Department, Ipswich Hospital, Heath Road, Ipswich, IP4 5PD UK; 3 Internal Medicine Department, University Hospital Complex of Pontevedra, Pontevedra, Spain; 4 Norwich Medical School, University of East Anglia and Norfolk and Norwich University Hospital, Norwich, UK; 5 Imperial College London, London, UK; 6 Centre for Nephrology, University College London, London, UK

**Keywords:** Lupus nephritis, Outcome, Renal transplant, SLE, Survival

## Abstract

Lupus nephritis (LN) is an important cause of morbidity and mortality in patients with systemic lupus erythematosus (SLE) often leading to end-stage renal failure (ESRF) and necessitating renal transplantation (rTp). Optimal timing of rTp in SLE patients with ESRF is uncertain and could potentially affect survival. We investigated the time spent on dialysis before rTp and survival following rTp in a cohort of SLE patients. Retrospective analysis of all adult SLE patients receiving rTp over a 40-year period (1975–2015) in two tertiary UK centres. Cox proportional hazard regression and receiver operator curves (ROC) were used to determine the risk associated with time on dialysis before rTp and other potential predictors. Forty patients (age 35 ± 11 years, 34 female, 15 Caucasian, 15 Afro–Caribbean and 10 South Asian) underwent rTp. During a median follow-up of 104 months (IQR 80,145), eight (20%) patients died and the 5-year survival was 95%. Univariate analysis identified time on dialysis prior to rTp as the only potentially modifiable risk predictor of survival with a hazard ratio of 1.013 for each additional month spent on dialysis (95% CI = 1.001–1.026, *p* = 0.03). ROC curves demonstrated that > 24 months on dialysis had an adverse effect with sensitivity of 0.875 and specificity 0.500 for death. No other modifiable predictors were significantly associated with mortality, indicating that time on dialysis had an independent effect. Increased time on dialysis pre-transplantation is an independent modifiable risk factor of mortality in this cohort of patients with lupus nephritis.

## Introduction

Systemic lupus erythematosus (SLE) is a heterogenous autoimmune rheumatic disease with particularly high prevalence in women of childbearing age [1]. The kidneys are often affected, with at least one-third of SLE patients developing overt renal disease, while 10–25% may reach end-stage renal failure (ESRF) requiring dialysis or kidney transplantation and 10–20% of patients die within 10 years [2]. Lupus nephritis (LN) remains one of the most common and severe manifestations of SLE. There are racial, ethnic and regional variations in the incidence, prevalence and prognosis of LN [3]. Specifically younger age (< 33 years), non-European ancestry and male gender (in some but not all series) were found to associate with earlier development of renal disease. Moreover, African–Caribbean, African–American and South Asian ethnicities usually have worse renal involvement when compared to other ethnic groups. Furthermore, Black and Hispanic patients with LN tend to have poorer prognosis and a higher risk of renal disease and mortality [4].

In those patients reaching ESRF, renal transplantation (rTp) has now become an accepted and preferred treatment. However, in the early era of renal transplantation, lupus patients were considered unfavourable candidates given an assumed risk of recurrent LN. Since 1975, however, when it was first suggested that the outcomes of transplant in SLE are comparable to non-SLE patients [5], there have been reports across the globe and in different ethnic populations that have shown low recurrence rates of LN in kidney transplant recipients [6–9]. Some studies, however, have raised concern regarding worse graft and patient survival of SLE patients when compared to other patient groups (e.g. patients with diabetes), with unfavourable comparative outcomes, especially for the recipients of deceased donors [10]. Nonetheless, there is a relative paucity of data in the literature regarding long-term outcomes of patients with LN and renal transplantation. Specifically, although in other patient cohorts undergoing renal transplantation the time spent on dialysis prior to the transplantation has been studied, showing that the longer a patient spends on dialysis, the worse the overall survival after the transplantation [11–13], this has not been specifically investigated for lupus patients. As time spent on dialysis before the transplantation can be a potentially modifiable factor, it is important to identify whether this is indeed a risk predictor in the lupus patients and whether there is a “safe maximum” time on dialysis before transplantation. We therefore investigated the long-term survival of patients with LN receiving rTp and the prognostic effect of the time spent on dialysis pre-transplant in our cohort of LN patients from two major London hospitals followed up since 1975.

## Methods

This was a retrospective review of all adult SLE patients (aged > 18 years) from two major London, UK institutions: University College London Hospital (UCLH) and Royal Free Hospital (RFH), who developed renal failure and received a renal transplant over a 40-year period (1975–2015). Hospital notes, electronic records and correspondence from family physicians and physicians in other hospitals were reviewed. All patients with SLE and related end-stage renal failure (defined as the need of chronic dialysis therapy or kidney transplantation due to primarily lupus nephritis) and who required renal transplantation from January 1975 to December 2015 were included in this study. In all patients, 6 months of disease quiescence was required prior to transplantation to be included. All patients fulfilled four or more of the 1982 revised classification criteria for SLE of the American College of Rheumatology [14] and histological class of lupus nephritis was defined according to the International Society of Nephrology/Renal Pathology Society (ISN/RPS) 2003 classification system [15], applied retrospectively for the patients who had undergone transplantation prior to 2003.

Published modifiable and non-modifiable parameters possibly associating with survival were considered, as shown in Table 1, and recorded for this cohort. This study was a retrospective review of a long-term observational registry for which University College London does not require formal ethical permission.

**Table 1:** Modifiable and non-modifiable potential risk factors investigated. *APLS*, antiphospholipid syndrome, *MI* myocardial infarctions, *TIA* transient ischaemic attack, *SLE* systemic lupus erythematosus, *LN* lupus nephritis, *ESRF* end-stage renal failure

Modifiable risk factors	Non-modifiable risk factors
Time on dialysis	Gender
Dialysis type—haemodialysis vs peritoneal dialysis	Ethnicity
Donor source—cadaveric vs living	Age of SLE diagnosis
	Age of LN
	Age of ESRF
	Time between SLE and LN
	Time between LN and dialysis
	Diabetes mellitus (type 1 or 2)
	Hypertension
	Dyslipidaemia
	APLS
	Cardiac disease (MI, stroke, TIA)
	Decade of renal transplantation

The primary endpoint was patient death. Mortality was assessed from dedicated SLE-transplant clinics and also from the database at the Office on National Statistics, a dedicated national registry where all the deaths in UK are recorded.

### Statistical analysis

Continuous variables are presented as mean and standard deviation. Categorical variables are presented as numbers and percentages. Cox proportional hazard regression and receiver operating characteristic (ROC) curves are used to determine potential predictors. The cumulative survival curves are drawn using the Kaplan–Meier method. Patient characteristics are summarised and expressed as mean ± SD (if normally distributed) or otherwise median and interquartile range (IQR). Comparison between living and dead patients was undertaken using chi-square, *t* test and Mann-Whitney non-parametric *t* test. A *p* < 0.05 was considered significant. IBM SPSS version 22 (IBM Corp., Armonk, NY, USA) was used for statistical analysis.

## Results

A total of 361 patients with lupus nephritis were identified (155 from RFH and 206 from UCLH). During the 42-year period of follow-up, 121 progressed to ESRF and 40 of these patients received a renal transplant (eight patients had been seen in both hospitals and included in the hospital where they were first seen). The patient characteristics and demographics are presented in Table 2.

**Table 2:** Demographic, clinical and histological features of the patients. *SLE* systemic lupus erythematosus, *rTp* renal transplantation, *ESRF* end-stage renal failure, *LN* lupus nephritis

Demographic characteristics	Patient (*n* = 40)
Gender/female	34
Ethnicity	
Caucasian	15
Black	15
Asian	10
Age at SLE diagnosis	21.1 ± 9.2
Age at ESRF	31.6 ± 10.4
Age at rTp	35.5 ± 11.0
Time on dialysis (months)	43 (13–49)
Time of follow-up (months)	104 (80–145)
Type IV LN	18
Donor source/cadaveric	22
Graft failure	9

Mean age at transplantation was 36 ± 11 years, and 34 (85%) were female. The self-reported ethnic distribution was similar to that seen in the general lupus cohort of the two hospitals, with 15 Caucasian (37.5%), 15 Afro–Caribbean (37.5%), and 10 South Asian (25.0%) undergoing rTp. Five patients were re-transplanted (two patients received a total of two transplants, and one patient received a total of three transplants). Follow-up time was initiated after the first transplant. Two of our patients (5%) had pre-emptive transplantation and the dialysis time for them was included as zero.

During a median follow-up of 104 months (IQR 80,145) 8 (20%), patients died (Table 3) and the 5-year survival was 95% which appeared similar across all decades (Table 4).

**Table 3:** Comparison of clinical demographics between patients who survived and who died after the renal transplantation

	Alive (*n* = 32)	Dead (*n* = 8)	*p* value
Gender/female	26	8	0.32
Age at lupus diagnosis (years)	21 ± 10	22 ± 9	0.77
Age LN	26 ± 8	26 ± 9	0.97
Age at ESRF	31 ± 9	33 ± 15	0.73
Age at renal transplantation (years)	36 ± 11	39 ± 14	0.34
Duration on dialysis prior to renal transplantations (months)	31 (12–39)	84 (68–90)	0.01
Ethnicity			
Caucasian	11 (34%)	4 (50%)	0.94
Black	15 (47%)	0	
Asian	6 (19%)	4 (50%)	
Type of dialysis, HD/PD*	17/9	3/3	0.64

*Eight patients required both PD and HD and therefore not included in the direct comparison between PD and HD. However, even when compared with PD or HD, there was no evidence that those who required both types of dialysis have worse outcome (*p* = 0.89)

*LN* lupus nephritis, *ESRF* end-stage renal failure, *HD* haemodialysis, *PD* peritoneal dialysis

**Table 4:** Comparison of 5-year mortality according to the decade the transplant was received

Table comparing survival according to decade of transplantation			*p* value
Dialysis per decade—5-year survival	Patients per decade	Mortality	
1975–85	2	0/2	0.97
1985–95	3	1/3 (33%)	
1995–05	8	2/8 (40%)	
2005–15	27	0/24*	

*Three patients from the 2005–2015 decade are alive but have not completed 5 years out from the transplantation and hence are not included in the table, explaining why it is 0/24 in the last decade

Three patients (37.5%) died as a consequence of sepsis, two as a consequence of uraemic complications (25%), two secondary to malignancy (25%) and one secondary to ischaemic heart disease (12.5%). Using univariate Cox regression time on dialysis and the other potential predictors of survival were investigated. Univariate analysis only identified time on dialysis prior to rTp as a predictor of survival with a hazard ratio of 1.013 for each additional month spent on dialysis (95% CI = 1.001–1.026, *p* = 0.03). No other parameter reached statistical significance as shown in Table 5. In particular, gender (*p* = 0.44), ethnicity (*p* = 0.99), age at SLE diagnosis (*p* = 0.55), age at LN (*p* = 0.94), age at rTp (*p* = 0.43), time between SLE diagnosis and LN (*p* = 0.37), time between LN and dialysis (*p* = 0.54) or indeed any other clinical co-existing diagnosis; DM (*p* = 0.56), hypertension (*p* = 0.32), dyslipidaemia (*p* = 0.91) did not affect survival. There was no difference between the decade the transplant took place and the outcome (*p* = 0.71) but this should be interpreted with caution in view of the low number of rTp undertaken in the earlier decades. We also compared the length of time on dialysis prior to transplantation in the patients who received the transplant before or after 2000, which was not statistically different (*p* = 0.181). Therefore, these results suggest that the time on dialysis was the only independent modifiable risk factor associated with mortality, irrespective of the decade the transplantation took place.

**Table 5:** Univariate Cox proportional hazard modelling investigating the association of various parameters and mortality showing that only risk factor associated with prognosis was time on dialysis, with longer time on dialysis associated with worse prognosis. *SLE* systemic lupus erythematosus, *LN* lupus nephritis, *ESRF* end-stage renal failure, *rTp* renal transplantation, *PD* peritoneal dialysis, *HD* haemodialysis, *APLS* antiphospholipid syndrome, *MI* myocardial infarction, *TIA* transient ischaemic attack

Factor	*p* value	HR	95% CI
Time on dialysis/per month	0.031	1.013	1.001–1.026
Gender/male	0.442	0.038	0.001–161.3
Ethnicity	0.987	0.995	0.537–1.844
Age at SLE diagnosis	0.552	1.021	0.953–1.094
Age of LN	0.941	1.003	0.920–1.092
Age of ESRF	0.836	1.008	0.935–1.087
Age at rTp	0.431	1.026	0.963–1.092
Dialysis PD (vs HD)	0.764	0.706	0.073–6.862
Time between SLE and LN	0.373	0.996	0.987–1.005
Time between LN and dialysis	0.540	0.999	0.994–1.003
LN duration before dialysis	0.152	1.066	0.977–1.164
Type IV LN	0.398	2.533	0.294–21.82
Dialysis decade	0.712	0.872	0.420–1.807
Diabetes mellitus	0.561	0.038	0.001–2319
Hypertension	0.323	0.329	0.360–2.987
Dyslipidaemia	0.905	0.872	0.092–8.234
APLS	0.508	0.036	0.000–672.6
Cardiac disease (MI, stroke, TIA)	0.873	1.071	0.463–2.476
Donor source (living)	0.353	0.459	0.089–2.372
Graft failure post rTp	0.314	2.073	0.501–8.567

Nine patients had received mycophenolate mofetil (MMF)/tacrolimus combination only, with no previous AZA or cyclosporine use, with the other patients having used (azathioprine) AZA or cyclosporine at any stage. The nine patients who received MMF/tacrolimus only had an overall mortality of 11.1% compared to the patients who ever received AZA/cyclosporine who had a mortality of 22.5%, although this difference did not reach statistical significance (*p* = 0.45). Finally, there was also no difference between the type of dialysis undertaken pre-transplantation and whether it was haemodialysis or peritoneal dialysis *p* = 0.64.

**Fig. 1:**
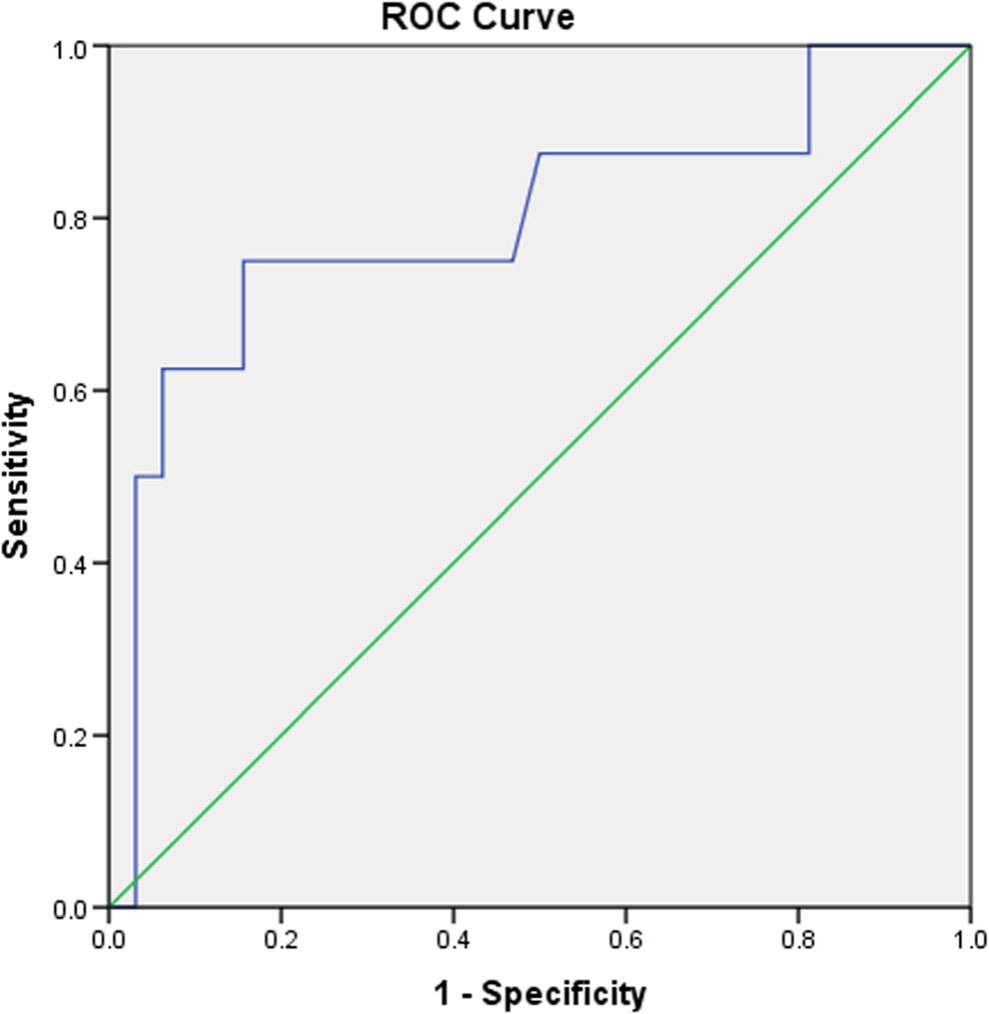
Receiver operator characteristic (ROC) curve between time on dialysis and survival. The area under the ROC curve was 0.795. Patients on dialysis for more than 24 months had a sensitivity of 0.875 and specificity of 0.500 to associate with mortality

Utilising specifically the time spent on dialysis before the transplantation, a ROC curve was used to calculate the optimal maximum time spent on dialysis prior to conferring an adverse outcome (Fig. 1) showing that > 24 months on dialysis had an adverse effect on survival, with an area under the ROC curve of 0.795, sensitivity of 0.875 and specificity 0.500 for death.

**Fig. 2:**
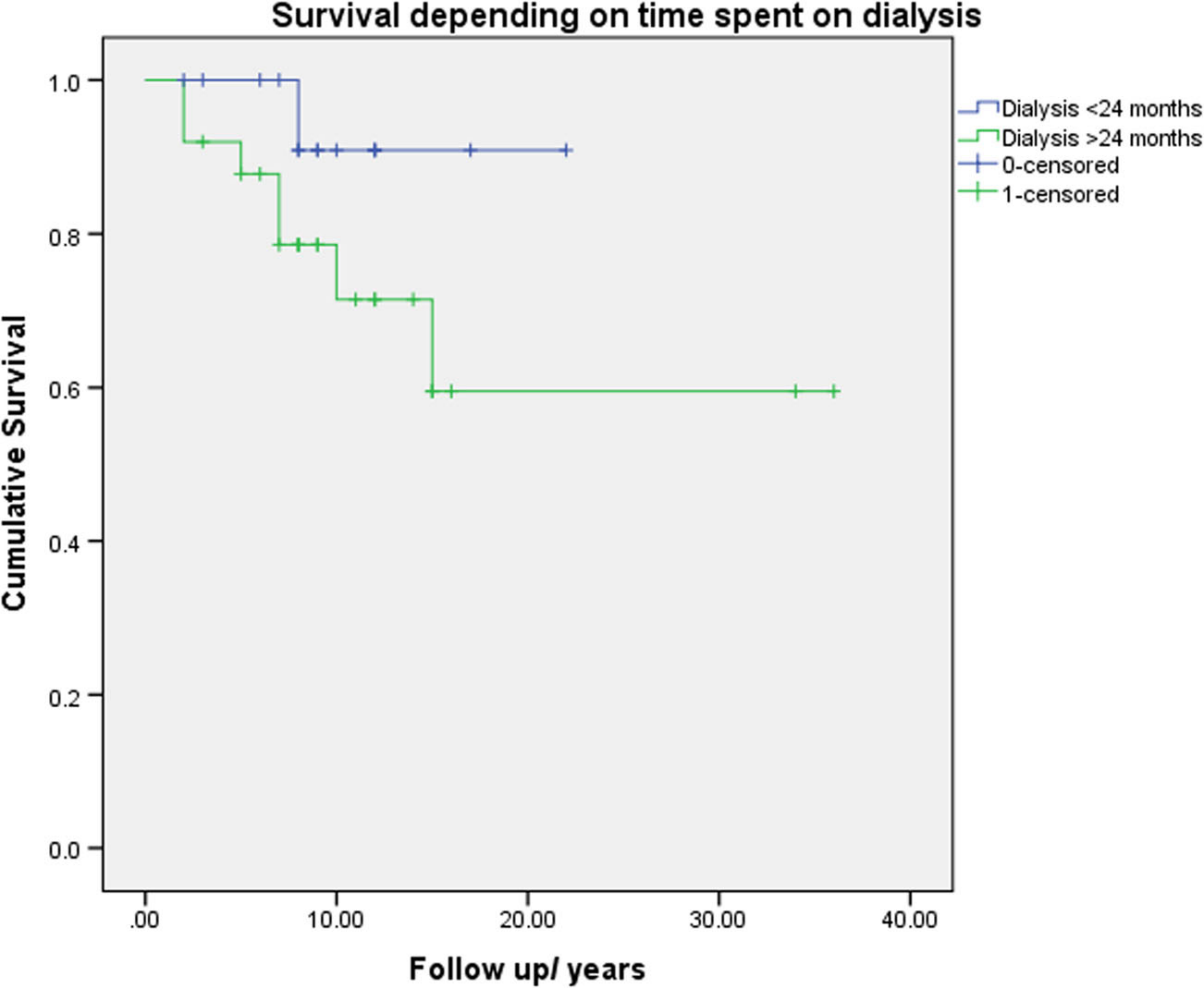
Kaplan–Meier estimator plot between patients who had less than 24 months of dialysis (blue line) or more than 24 months (green line), suggesting a trend of almost threefold risk of survival in those spending longer time on dialysis, HR 2.84 log rank *p* = 0.15

Utilising this dichotomous value, there was a 2.8-fold higher risk of mortality in those patients who spent longer than 24 months on dialysis using Kaplan–Meier curves (Fig. 2), although there was only a trend towards statistical significance seen (log rank *p* = 0.15). This supports the results from the Cox regression which showed that mortality was increased by 1.3% for every additional month on dialysis (or 15.6% for every additional year on dialysis) and that most likely if transplantation could be undertaken by 24 months on dialysis, or even earlier, it could be of benefit to the patients.

Although not the aim of our study, we also compared the overall survival of the patients with LN-related ESRF receiving transplantation vs the ones without transplantation. In total, 45/81 (56%) died in the non-transplanted patients compared with 8/40 (20%) of those who received at least one renal transplant (*p* = 0.0002). Although the superiority of renal transplantation in this context is well recognised [16], this result could have been confounded by higher burden of comorbidity in the patients not selected for transplantation.

## Discussion

Patients with lupus nephritis represent a complex cohort of patients which should be managed optimally to ensure longer-term survival. In the present study, we focused on time spent on dialysis pre-transplantation for renal nephritis as a potentially modifiable predictor of patient mortality, rather than predictor of graft failure. We also investigated other potential predictors of survival both modifiable and non-modifiable. We included patients going back to the early times of rTp in LN from 1975 and we present data on the longest reported follow-up period for a dedicated cohort of patients with LN undergoing renal transplantation. We identified a 5-year survival of 95% which is in line or better than other published studies [9, 10, 17]. Survival did not appear to differ in relation to the decade the rTp took place, although this should be considered within the context of the low numbers of rTp in the very early decades meaning that the study might have been underpowered to detect a small but clinically important difference.

The only variable that appeared to offer any prognostic association with mortality was time spent on dialysis prior to the transplant. For every additional month on dialysis, prognosis worsened by 1.3%. If patients exceeded a binary cut-off of 24 months on dialysis in our cohort, there was a suggestion that this conferred almost a threefold increase in mortality. No other factors appeared to affect mortality, as they did not reach significance in univariate analysis.

The optimal timing of transplantation in patients with LN reaching ESRF is not known, but this study would support earlier transplantation if feasible. This is similar to recent work which found that increased time on dialysis led to increased graft failure [9, 18]. Indeed, our cohort included two patients with pre-emptive transplantation and they both remain alive at 22 and 12 years respectively, supporting that the earlier benefits of rTp. Although our research identifies the cut-off of 24 months which could be used to prioritise rTp in LN patients, further larger and prospective studies are necessary to identify whether the time relationship to survival up to 24 months is a linear one or whether an even much earlier and possibly even pre-emptive transplantation should be recommended and incorporated in current guidelines.

### Limitations

Despite combining the data from two large institutions, we only had 40 patients to analyse, which is however in line or larger than other similar published studies [9, 17]. Our cohort also included a mixture of Caucasian, Afro–Caribbean and South Asian patients and we cannot necessarily extrapolate our results to patients from other ethnicities. Larger studies including multiple ethnicities will also allow further comparisons. Finally, despite a very long follow-up of 422 patient-years, only eight patients reached the study endpoint which may have reduced the identification of the impact of other potentially predictive variables, for example sex and presence of antiphospholipid syndrome in particular, which had a wide confidence interval in our results. In addition, although we could only undertake univariate analysis due to the small number of outcomes, this still allows us to accurately identify individual predictors and trends towards mortality. Especially as only the time on dialysis was significant, with patients spending similar times on dialysis throughout the 40-year period, we can be confident that this was not influenced or affected by other parameters. Nonetheless, we propose that ultimately, multicentre interventional studies are required to provide adequate power to address this specific question.

## Conclusion

In conclusion, in this long-term follow-up study of patients with SLE and rTp from two large institutions spanning across four decades, we identified that the only potential modifiable factor to improve survival was reducing the time on dialysis prior to transplantation. This finding should be validated in larger multicentre studies and help identify the optimal timing of transplantation in LN following ESRF whether on dialysis or pre-emptively [19].
